# A decision tree to assess short-term mortality after an emergency department visit for an exacerbation of COPD: a cohort study

**DOI:** 10.1186/s12931-015-0313-4

**Published:** 2015-12-22

**Authors:** Cristóbal Esteban, Inmaculada Arostegui, Susana Garcia-Gutierrez, Nerea Gonzalez, Iratxe Lafuente, Marisa Bare, Nerea Fernandez de Larrea, Francisco Rivas, José M. Quintana, for the IRYSS-COPD group

**Affiliations:** Servicio de Neumologia, Hospital Galdakao-Usansolo, Barrio Labeaga s/n. 48960, Galdakao, Bizkaia Spain; Departamento de Matemática Aplicada y Estadística e Investigación Operativa, Universidad del País Vasco UPV/EHU, Leioa, Bizkaia Spain; Unidad de Investigación, Hospital Galdakao-Usansolo, Galdakao, Bizkaia Spain; Unidad de Epidemiología Clínica, Corporacio Parc Tauli, Barcelona, Spain; Departamento de Salud, Madrid, Spain; Unidad de Investigación, Hospital Costa del Sol, Malaga, Spain; Red de Investigación en Servicios Sanitarios y Enfermedades Crónicas (REDISSEC), Galdakao, Bizkaia Spain; Basque Center for Applied Mathematics - BCAM, Bilbao, Spain

**Keywords:** COPD, Decision tree, Short-term mortality

## Abstract

**Background:**

Creating an easy-to-use instrument to identify predictors of short-term (30/60-day) mortality after an exacerbation of chronic obstructive pulmonary disease (eCOPD) could help clinicians choose specific measures of medical care to decrease mortality in these patients. The objective of this study was to develop and validate a classification and regression tree (CART) to predict short term mortality among patients evaluated in an emergency department (ED) for an eCOPD.

**Methods:**

We conducted a prospective cohort study including participants from 16 hospitals in Spain. COPD patients with an exacerbation attending the emergency department (ED) of any of the hospitals between June 2008 and September 2010 were recruited. Patients were randomly divided into derivation (50 %) and validation samples (50 %). A CART based on a recursive partitioning algorithm was created in the derivation sample and applied to the validation sample.

**Results:**

Two thousand four hundred eighty-seven patients, 1252 patients in the derivation sample and 1235 in the validation sample, were enrolled in the study. Based on the results of the univariate analysis, five variables (baseline dyspnea, cardiac disease, the presence of paradoxical breathing or use of accessory inspiratory muscles, age, and Glasgow Coma Scale score) were used to build the CART. Mortality rates 30 days after discharge ranged from 0 % to 55 % in the five CART classes. The lowest mortality rate was for the branch composed of low baseline dyspnea and lack of cardiac disease. The highest mortality rate was in the branch with the highest baseline dyspnea level, use of accessory inspiratory muscles or paradoxical breathing upon ED arrival, and Glasgow score <15. The area under the receiver-operating curve (AUC) in the derivation sample was 0.835 (95 % CI: 0.783, 0.888) and 0.794 (95 % CI: 0.723, 0.865) in the validation sample. CART was improved to predict 60-days mortality risk by adding the Charlson Comorbidity Index, reaching an AUC in the derivation sample of 0.817 (95 % CI: 0.776, 0.859) and 0.770 (95 % CI: 0.716, 0.823) in the validation sample.

**Conclusions:**

We identified several easy-to-determine variables that allow clinicians to classify eCOPD patients by short term mortality risk, which can provide useful information for establishing appropriate clinical care.

**Trial registration:**

NCT02434536.

**Electronic supplementary material:**

The online version of this article (doi:10.1186/s12931-015-0313-4) contains supplementary material, which is available to authorized users.

## Background

Outcomes as long-term mortality and short-term mortality are frequently used in studies of mortality following hospitalization for an exacerbation of COPD (eCOPD) [1, 2]. In long-term follow-up, mortality is likely more influenced by the general characteristics of the patient’s COPD than the severity of the eCOPD that triggered the admission. Short-term mortality, usually defined as mortality occurring less than 90 days after presentation to a hospital with an eCOPD, usually includes in-hospital mortality, and factors related to the severity of the exacerbation likely play more important role in the short term rather than in the mid or long term follow-up.

CART models have been used in various disciplines and diseases [3, 4]. CART analysis has also been used to predict 5-year mortality among patients with stable COPD using five easily obtained parameters in clinical practice with good predictive capacity compared to other prognostic multidimensional based instruments [5]. CART has also been used to predict short-term or long-term mortality and the need for mechanical ventilation among patients with acute exacerbations of COPD [6].

Many studies evaluating short-term outcomes in eCOPD patients have focused on the 90-day period following the episode. We focused on a shorter period after the index event (30/60 days) in an effort to find factors more closely related to the eCOPD event and differences in predictive factors for both mortality periods. Few studies have taken this approach, even though important outcomes such as mortality and readmissions occur during this period. The aim of our study was to develop and validate a CART to predict 30 and 60-days mortality following an emergency department (ED) evaluation for an eCOPD. Identifying factors related to mortality during this period could provide valuable information about the appropriate clinical care for these patients.

## Methods

We used data collected as part of the Investigacion en Resultados y Servicios de Salud COPD Appropriateness (IRYSS-COPD) for this investigation. Methods of the IRYSS-COPD Study have been described in detail elsewhere [7]. In brief, this prospective cohort study included patients with an eCOPD attending the emergency departments (ED) of 16 hospitals in Spain between June 2008 and September 2010. All patients were informed of the goals of the study and invited to voluntarily participate in it. Patients who agreed to participate provided written consent. All information was kept confidential.

Patients were eligible for the study if they presented to the ED with symptoms consistent of an eCOPD. COPD was confirmed if the patient had a forced expiratory volume in 1 second/forced vital capacity (FEV_1_/FVC) quotient <70 %. Exacerbation was defined as an event in the natural course of a patient’s COPD characterized by a change in baseline dyspnea, cough, and/or sputum that was beyond normal day-to-day variations and that may have warranted a change in medication or treatment [8]. For patients already known to have been diagnosed of COPD, the closest sprirometry data to the ED index visit performed at a time when the patient was stable, and not longer than 6 months, was taken as reference. When COPD was newly diagnosed during the ED visit, to be included in the study, the patient had to have COPD confirmed by spirometry within 60 days after the index episode at a time when he or she was stable [9]. In those cases, that spirometry was taken as reference. No spirometry data was recorded from the ED nor during the admission to the hospital.

Gold [9] patients were excluded from the study if, at the time they were seen in the ED, the eCOPD was complicated by a comorbidity such as pneumonia, pneumothorax, pulmonary embolism, lung cancer, or left cardiac failure. Other exclusion criteria included a diagnosis of asthma, extensive bronchiectasis, sequelae of tuberculosis, pleural thickening, or restrictive diseases. Patients who did not wish to participate were also excluded.

### Data collected

Data collected upon arrival in the ED included socioeconomic information, clinical data at baseline, presence of pathologies recorded in the Charlson Comorbidity Index [10], and information about the eCOPD event such as arterial blood gases, respiratory rate, dyspnea and consciousness level measured by the Glasgow Coma Scale [11].

For patients admitted to the hospital, we collected additional data from the medical record and from a direct interview with the patient on the first day after admission and also on the day of discharge. We asked all patients to tell us about their physical activity, general health, and dyspnea level when they were in a stable condition before the eCOPD and 24 hours after being admitted to the hospital or discharged from the ED to home. We used the Medical Research Council (MRC) breathlessness scale [12] to measure baseline dyspnea.

For all patients with known COPD, additional variables collected from medical records included baseline severity of COPD as measured by FEV_1_; hospital admissions for eCOPD during the previous 12 months; baseline therapy and the presence of conditions needed to determine the Charlson Comorbidity Index.

Reviewers were trained to identify pertinent data and a precise manual was developed to help reviewers collect the data.

Mortality at 30/60 days after the index ED index was determined by consulting medical records, regional electronic databases and the national registry of mortality.

### Statistical analysis

The outcomes variables were defined as mortality within 30 days or 60 days of the index ED visit for the eCOPD. Patients were randomly divided into a derivation sample (50 %) and a validation sample (50 %). Derivations sample was used in order to develop the CART, whereas validation sample was used to validate the results obtained from the previously derived CART. Both samples were described using means and the standard deviations for continuous variables and the number of cases and percentages for categorical variables. Differences between the derivation and the validation samples were tested for the distribution of each variable using the *t*-test for continuous variables and the chi-square test for categorical variables. Missing data on baseline level of dyspnea (MRC breathlessness scale) were considered as a separate category from other data. Justification for the inclusion of missing dyspnea data was based on the fact that patients without a measurement of baseline dyspnea were significantly different from patients with lower levels of breathlessness (MRC categories 1 to 4 [*p* < 0.0001]) but not from those with the most severe breathlessness (MRC category 5 [*p* = 0.63]).

A CART based on a recursive partitioning algorithm was created in the derivation sample [13] to identify 30-day mortality risk factors with the highest discriminative power. The goal was to identify the variables and partition point that optimally separate low-risk patients from high-risk patients. To internally validate the risk of 30-day mortality derived from the regression tree, we used bootstrap resampling with *N* = 2,000 repetitions and estimated 95 % confidence intervals (95 % CI) [14]. We report the median of these 2,000 repetitions as the parameter estimate and the 2.5 and 97.5 percentiles as the 95 % CIs.

To make the CART more user friendly, we simplified the resulting algorithm into a manageable number of risk classes based mainly on the estimated risk of 30-day mortality. We applied the risk classification derived from the derivation sample to the validation sample. The Cochran-Armitage trending statistic was performed to assess whether classification provided by the CART could differentiate low-risk patients from high-risk patients in a fashion of graded response based on the level of risk present.

The 30-day mortality derived and validated classification tree was applied to 60-day mortality. Additional variables to improve the 30-day mortality risk prediction to 60-day mortality risk prediction were selected from the univariate analysis and added to the CART analysis in order to get the best prediction tree for 60-day mortality.

Model discrimination of the trees and the risk categories was assessed by the area under the receiver operating curves (AUC).

Effects were considered statistically significant at α = 0.05. All statistical analyses were performed using SAS for Windows© version 9.1, except for the development and validation of the regression tree, which was built using R version 2.14.

## Results

**Fig. 1:**
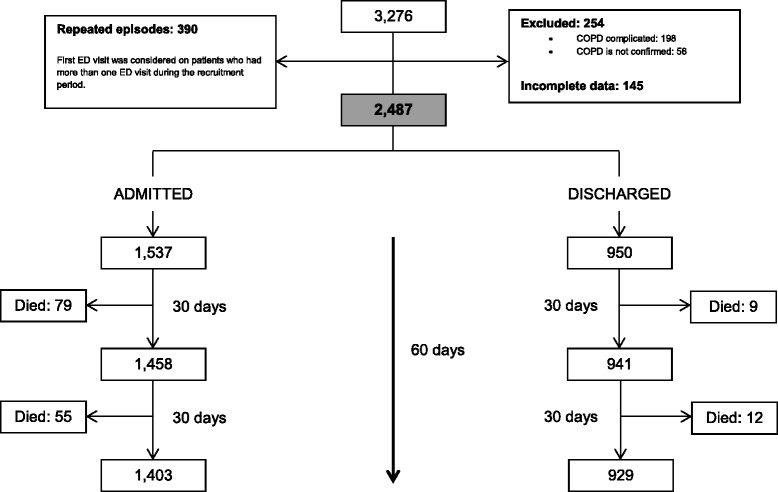
Flow-chart of the sample

A total of 2487 patients were enrolled in the study. (Figure 1) they were divided into the derivation sample (1252 patients) and the validation sample (1235 patients). The only difference between the two samples was use of accessory inspiratory muscles at ED admission, which was higher in the derivation sample (more information in Additional file 1: eTable S1).

**Fig. 2:**
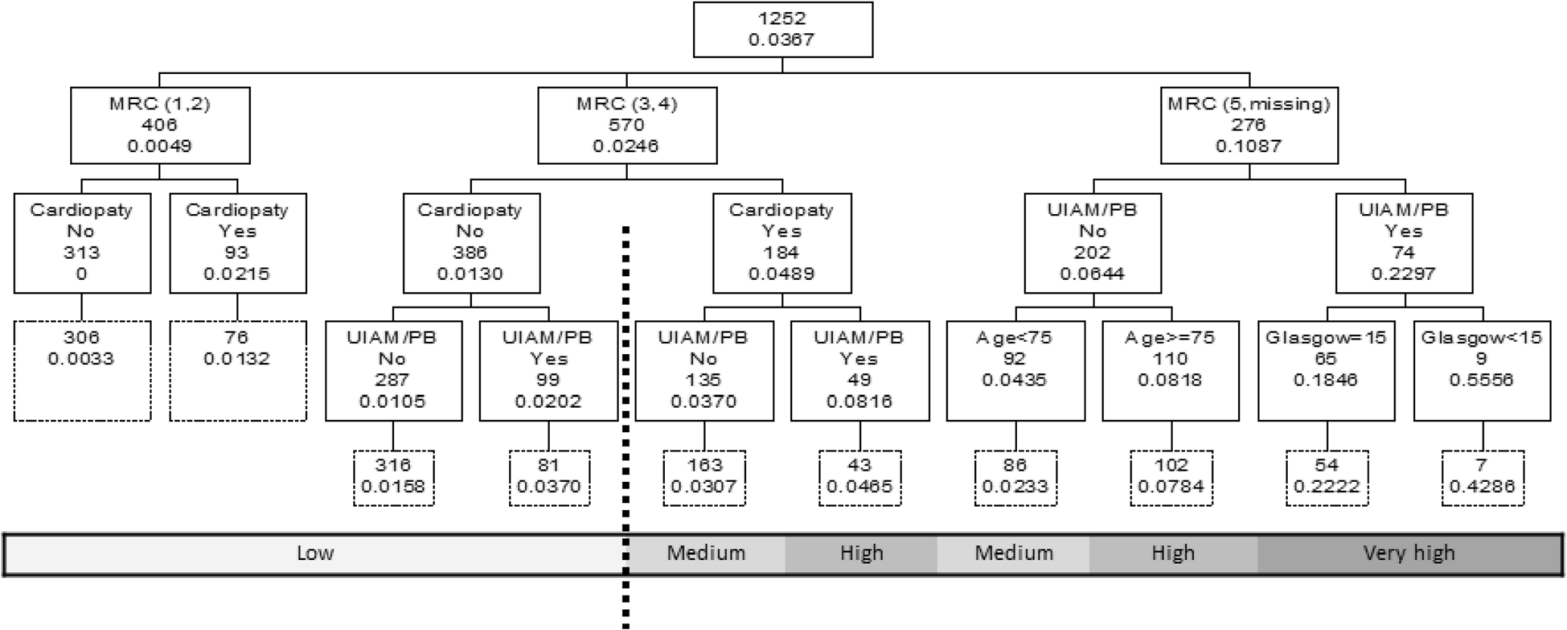
Results of the CART analysis for 30-day mortality in the derivation sample. Application to the validation sample is shown below each node in dashed boxes. Each node shows the classification variable plus the number of subjects and the estimated probability of 30-day mortality on that node. Estimated one-month mortality risk has been categorized in low, medium, high and very high as show below the tree. Dashed vertical line shows the cut-off point for dichotomization of estimated mortality risk looking for optimal sensitivity-specificity combination in the derivation sample, leading to a sensitivity of 0.651 and a specificity of 0.848. UIAM = Use of inspiratory accessory muscle; PB = Paradoxical breathing; MRC = MRC breathlessness scale

Factors associated with mortality are shown in Table 1. Based on results of the univariate analysis, we included these significant variables in the splitting process of building the classification tree for 30-day mortality: the MRC baseline dyspnea scale, cardiac disease, presence of UAIM or PB at ED admission, age, and Glasgow Coma Scale score. The CART created for 30-day mortality with data from the derivation cohort is shown in Fig. 2. No patients with a low level of dyspnea at baseline (MRC breathlessness level 1–2) and without cardiac disease died in the 30-day follow-up period. In contrast, 55 % of patients with the most extreme baseline dyspnea (MRC breathlessness level 5), UAIM/PB at ED admission, and a Glasgow Coma Scale score <15 died during the 30-day follow-up period. Among patients with MRC level 5 dyspnea at baseline, those without UAIM/PB who were less than 75 years old had a mortality rate of 4 %. The AUC in the derivation sample was 0.835 (95 % CI: 0.783, 0.888) and 0.794 (95 % CI: 0.723, 0.865) in the validation sample.

**Table 1:** Univariate analysis performed in the derivation sample (*n* = 1252)

	30-day mortality		60-day mortality	
	No	Yes	No	Yes
	1206 (96.3 %)	46 (3.7 %)	1173 (93.7 %)	79 (6.3 %)
Age^a^	72.5 (9.8)	77.7 (8.6)*	72.3 (9.8)	77.3 (8.9)*
Sex (Male)	1105 (91.6)	42 (91.3)	1075 (91.7)	72 (91.1)
Baseline FEV1 %		**		
≥50	42 (4.2)	0 (−)	40 (4.1)	2 (3.0)
30 < FEV1 % < 50	295 (29.4)	6 (14.6)	286 (29.2)	15 (22.7)
≤30	666 (66.4)	35 (85.4)	652 (66.7)	49 (74.2)
Charlson Comorbidity Index^a^	2.22 (1.52)	3.11 (2.23)*	2.19 (1.50)	3.15 (2.00)*
>2	374 (31.0)	24 (52.2)**	351 (29.9)	47 (59.5)*
Diabetes mellitus	238 (19.8)	19 (41.3)*	230 (19.7)	27 (34.2)**
Cardiopathy	345 (28.6)	24 (52.2)**	327 (27.9)	42 (53.2)*
Previous LT-DOT or NIMV at home	380 (31.5)	34 (73.9)*	367 (31.3)	47 (59.5)*
Number of previous admissions due to eCOPD	0.85 (1.38)	1.04 (1.28)	0.84 (1.38)	1.08 (1.37)
0-1	970 (80.4)	35 (76.1)	445 (80.6)	60 (76.0)
2	103 (8.5)	2 (4.4)	101 (8.6)	4 (5.1)
≥3	133 (11.0)	9 (19.6)	127 (10.8)	15 (19.0)
Glasgow Coma Score – <15	32 (2.7)	7 (15.2)*	30 (2.6)	9 (11.4)*
Heart rate upon ED arrival (≥120)	104 (8.6)	8 (17.4)	99 (8.4)	13 (16.5)
Use of inspiratory accessory muscle upon ED arrival	256 (21.2)	23 (50.0)*	252 (21.5)	27 (34.2)**
Paradoxical breathing upon ED arrival	52 (4.3)	9 (19.6)*	48 (4.1)	13 (16.5)*
pH upon ED arrival		*		**
≥7.35	986 (88.6)	30 (68.2)	958 (88.4)	58 (79.5)
7.26-7.34	106 (9.5)	8 (18.2)	105 (9.7)	9 (12.3)
<7.26	21 (1.9)	6 (13.6)	21 (1.9)	6 (8.2)
PCO2 upon ED arrival		*		*
≤45	613 (58.5)	10 (23.3)	596 (58.4)	27 (38.0)
46-55	233 (22.2)	14 (32.6)	226 (22.2)	21 (29.6)
56-65	111 (10.6)	9 (20.9)	109 (10.7)	11 (15.5)
>65	91 (8.7)	10 (23.3)	89 (8.7)	12 (16.9)
MRC breathlessness scale		*		*
1-2	404 (33.5)	2 (4.4)	401 (34.2)	5 (6.3)
3	227 (18.8)	3 (6.5)	220 (18.8)	10 (12.7)
4	329 (27.3)	11 (23.9)	319 (27.2)	21 (26.6)
5	119 (9.9)	19 (41.3)	109 (9.3)	29 (36.7)
missing	127 (10.5)	11 (23.9)	124 (10.6)	14 (17.8)

^a^Represented as mean (sd)

Statistically significant differences between deceased and not deceased patients at each period of time for each characteristic are shown, *stands for *p* < 0,001 and **stands for 0.05 < *p* < 0.001

**Fig. 3:**
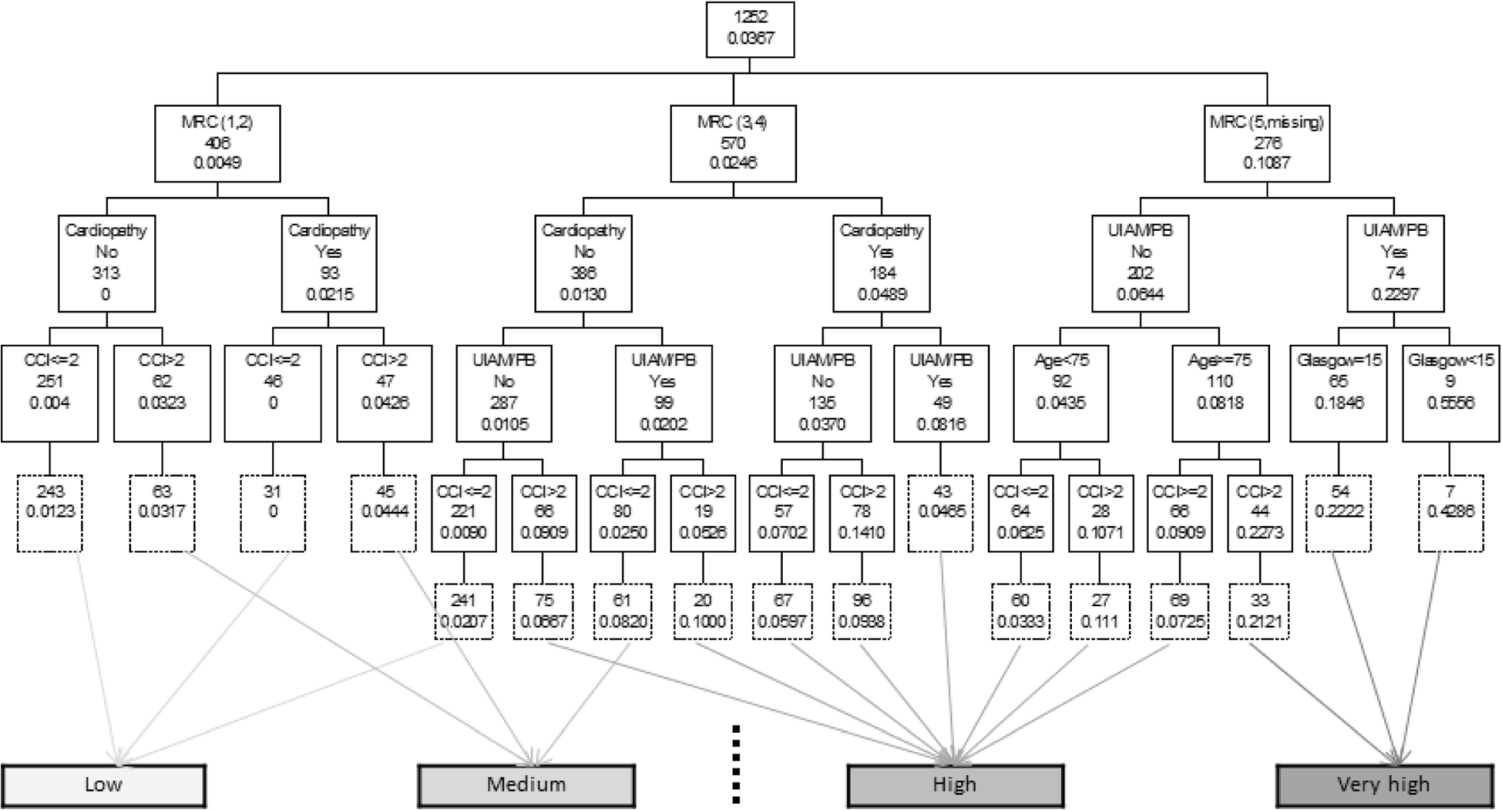
CART model for 60-day mortality in the derivation sample. Application to the validation sample is shown below each node in dashed boxes. Each node shows the classification variable plus the number of subjects and the estimated probability of 60-day mortality on that node. Estimated 60-day mortality risk has been categorized in low, medium, high and very high as show below the tree. Dashed vertical line shows the cut-off point for dichotomization of estimated mortality risk looking for optimal sensitivity-specificity combination in the derivation sample, leading to a sensitivity of 0.662 and a specificity of 0.823. UIAM = Use of inspiratory accessory muscle; PB = Paradoxical breathing; MRC = MRC breathlessness scale; CCI = Charlson Comorbidity Index

When applied the original 30 days mortality CART to 60 days mortality the AUC was 0.774, (95 % CI: 0.723, 0.826) in the derivation and 0.73, (95 % CI: 0.671, 0.790) in the validation sample. The Charlson Comorbidity Index (>2/<=2) was identified as the only significant variable that added to that the 30-days tree increases the CART prediction for mortality risk at 60-days (Fig. 3). Then, the AUC in the derivation sample improves to 0.817 (95 % CI: 0.776, 0.859) and to 0.770 (95 % CI: 0.716, 0.823) in the validation sample.

Using data from the derivation sample, the CART tree created four short-term mortality risk classes. This risk classification was validated in the validation (Table 2). The Cochran-Armitage test showed a statistically significant trend in both samples (*p* < 0.0001). Risk classes were also established and validated for 60 days mortality. Though heart rate was flagged as a predictor by some of the CART models, the use of UAIM/PB provided more reliable data. Additional analysis showed that heart rate and UAIM were correlated. Patients without cardiac disease and without UAIM/PB at ED evaluation had a mean heart rate of 93.3 (17.9) compared to a mean heart rate of 102.1 (21.4; *p* = 0.0005) among those who demonstrated UAIM/PB at ED evaluation.

**Table 2:** Distribution of subjects by estimated 30/60-day mortality and stratified risk group in both samples

	Derivation sample (1252)		Validation sample (1235)	
30-day mortality				
Risk group (Fig. 1)	No (1206)	Yes (36)	No (1193)	Yes (42)
Low	785 (99.1)	7 (0.9)	769 (98.7)	10 (1.3)
—	—	—	—	—
Medium	218 (96.0)	9 (4.0)	243 (97.2)	7 (2.8)
High	146 (91.8)	13 (8.2)	135 (93.1)	10 (6.9)
Very high	57 (77.0)	17 (23.0)	46 (75.4)	15 (24.6)
AUC	0.808 (0.742 – 0.873)		0.767 (0.686 – 0.847)	
60-day mortality				
Risk group (Fig. 2)	No (1173)	Yes (79)	No (1159)	Yes (76)
Low	515 (99.4)	3 (0.6)	507 (98.4)	8 (1.6)
Medium	183 (96.8)	6 (3.2)	160 (94.7)	9 (5.3)
—	—	—	—	—
High	387 (90.6)	40 (9.4)	424 (92.8)	33 (7.2)
Very high	88 (74.6)	30 (25.4)	68 (72.3)	26 (27.7)
AUC	0.798 (0.757 – 0.838)		0.744 (0.691 – 0.898)	

Dashed horizontal lines shows the cut-off points for dichotomization of estimated mortality risk looking for optimal sensitivity-specificity combination in the derivation sample, leading to a sensitivity of 0.651 and a specificity of 0.848 for 30-day mortality risk and a sensitivity of 0.662 and a specificity of 0.823 for 60-day mortality risk

**Fig. 4:**
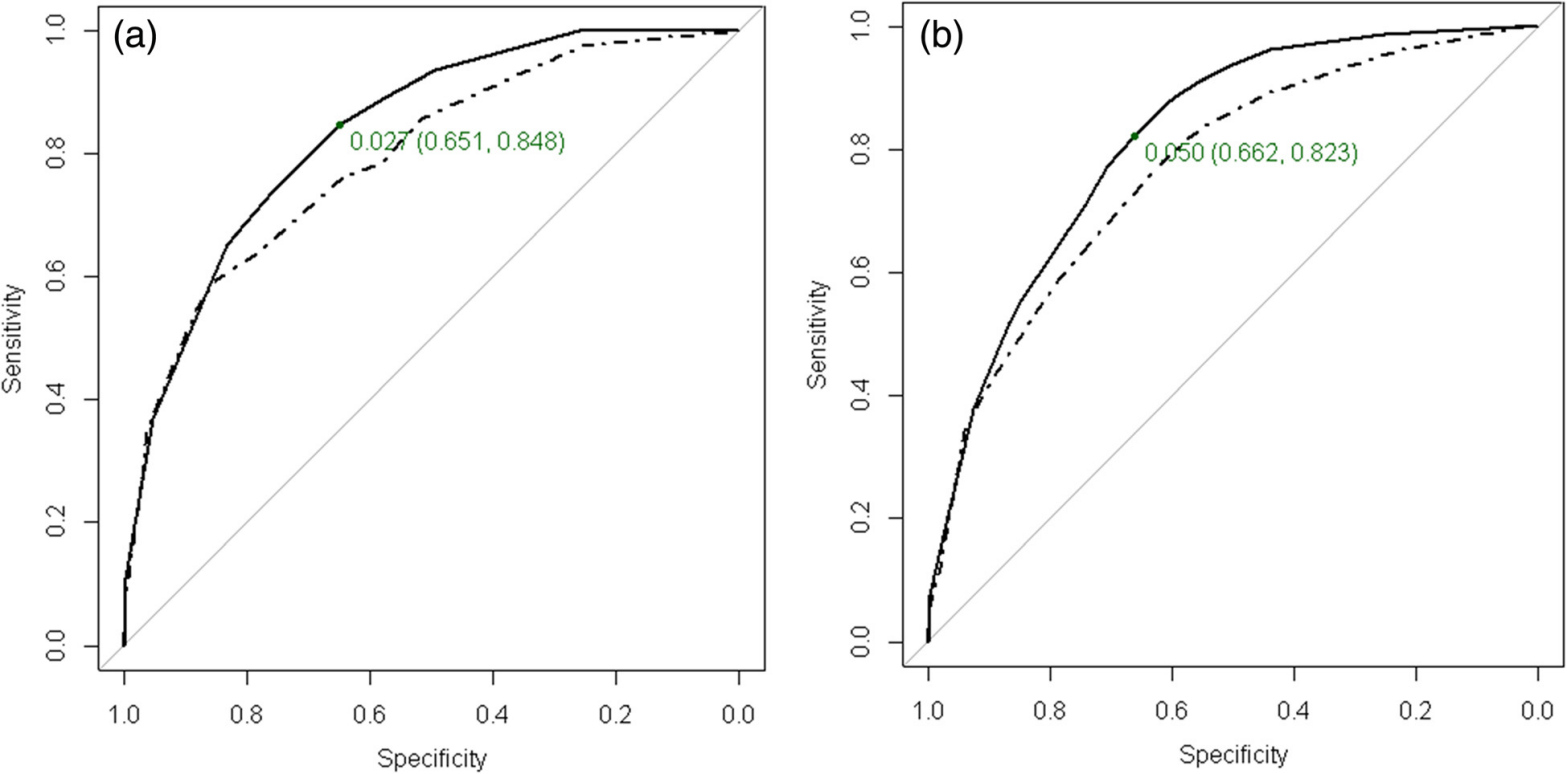
Roc curve for risk 30-day (**a**) and 60-day (**b**) mortality predicted by the CART analyses. Solid line applies for derivation sample and dashed line applies for validation sample. The cut-off point of estimated risk dichotomization for optimal sensitivity-specificity combination for derivation sample is shown in grey with the corresponding sensitivity and specificity values. **a** 30-day mortality: AUC = 0.835 and 95 % confidence interval is (0.783, 0.888) for derivation sample and AUC = 0.794 and 95 % confidence interval is (0.723, 0.865) for validation sample. **b** 60-day mortality: AUC = 0.817 and 95 % confidence interval is (0.776, 0.859) for derivation sample and AUC = 0.770 and 95 % confidence interval is (0.716, 0.823) for validation sample

More detailed results on internal bootstrap validation are shown in Additional file 1: eFigure S1 and Additional file 1: eTable S2. AUC for the CARTs decision trees in the derivation and validation samples for both mortality outcomes are presented in Fig. 4.

## Discussion

Dyspnea at baseline was the main variable associated with 30-day mortality after an ED evaluation for eCOPD. It provided the first branch of the CART. The next branches included the presence of cardiac disease, age, UAIM/PB, and the Glasgow Coma Scale score.

In our cohort, 3.6 % of patients died within 30 days of the index eCOPD. In similar studies, mortality during this period was 1 % [15] and 2.1 % [16]. At 60-day after the event mortality rate almost doubled the rate.

The variable that established separation in the first branch of the CART was baseline dyspnea. Among patients with stable COPD, dyspnea has been identified as a more important prognostic factor than pulmonary function (FEV1) [17, 18]. In two studies of in-hospital mortality among eCOPD patients, baseline dyspnea was independently associated with mortality [19, 20]. In contrast, a study evaluating long-term mortality (4 years) following hospitalization for an eCOPD found that dyspnea at baseline was associated with mortality in the univariate analysis but not in the multivariate analysis [21]. Therefore one variable reflecting patients’ baseline situation, dyspnea, was a very important predictor of in-hospital and very short-term mortality after an eCOPD requiring hospital evaluation. In our study, patients with a level 5 on the MRC breathlessness scale had higher mortality (18 %–55 % depending on the absence or presence of neurological impairment). In contrast, patients with level 1–2 dyspnea at baseline had low rates of mortality (0 %–2 %).

Cardiac comorbidity was the second-level key factor in the CART. In analyzing mortality 90 days after discharge for an eCOPD, Almagro et al. identified age, FEV1 according the GOLD classification, number of previous hospitalizations for COPD, use of home oxygen therapy, higher functional dependence, and comorbidities according Charlson comorbidity index as factors related to mortality [22]. In our study, cardiac diseases had a greater impact on 30-day mortality than comorbidities in general. Donaldson et al. showed that in a short period of time (1–5 days) after an eCOPD treated with antibiotics and systemic steroids, the risk of myocardial infarction significantly increased [23]. In our cohort, 2 % of patients with little breathlessness at baseline (MRC levels of 1–2) and cardiac disease died within 30 days compared to 0 % who did not have cardiac disease. A similar pattern was seen among patients with more severe breathlessness (MRC level 3–4), with mortality rates of 5 % among those with cardiac disease and 1 % among those without it. Cardiac disease and COPD share smoking and low-grade chronic inflammation as potential factors that could explain their coexistence.

In a prospective study of patients admitted for an eCOPD, 10 % had increases in their troponin T levels [24]. In this group, even a modest increase in this biomarker was associated with an increase in long-term mortality, especially in the first year after the episode [24]. The mortality rate increases further when troponin T elevations are associated with tachycardia (>100 beats/min) [25]. Thirty-day mortality among patients hospitalized for an eCOPD has also been independently associated with elevated levels of NT-proBNP or troponin T; when both NT-proBNP and troponin T were elevated, mortality was 15-fold higher than among patients with normal values [26].

The design of our study did not modify the usual clinical practice, so troponin T and NT-proBNP were measured at the discretion of the treating clinician. Thus, we were not able to measure the impact of these markers.

Roche et al. [19], using a similar study as ours but focused on in-hospital mortality, established a model that included age, baseline dyspnea, and a severity index that include UAIM and neurological impairment. This model is very similar to the variables included in our CART. However, there are some differences. First, our CART included cardiac disease as a key comorbidity in the final outcome. Second, the variables included in our CART did not affect every patient. In fact, neurologic impairment (Glasgow Coma Scale score <15) was included in only one branch which, added to the UAIM/PB and with a dyspnea grade of 5, was associated with the highest mortality rate of the whole cohort, 55 %.

A low Glasgow Coma Scale score has been associated with increased mortality in ICU-admitted eCOPD patients [27], but not in short-term (30-day) follow-up.

Variables such as severity of airway obstruction and previous hospitalizations for eCOPD have been cited as factors related to eCOPD mortality. In our study, though, they were not identified as prognostic variables for 30-day mortality following ED evaluation for an eCOPD.

Once the CART developed for 30-day mortality risk prediction was applied to 60-day mortality risk prediction predictive ability decreases. However, a new variable took a relevant role in almost every branch of the decision tree, number of comorbidities as measured by the Charlson Comorbidity–index, improving the predictive ability of the tree for 60-day mortality risk prediction. Comorbidities have been considered an important mortality predicting factor [22] even has been included in some multidimensional prognosis index. [16, 28] This implies that factors related to mortality after such a hospitalization change in a short -time, reaching progressively more importance some aspect of the general clinical condition of the patient as comorbidities are. This is a key issue when considering the global treatment and follow-up for these patients.

Our decision tree was built by recursive partitioning using CART. One important advantage of CART over linear and additive models is that it does not require parametric specification of the nature of the relationship between predictors and outcome. In practice, this means that the assumption of linearity, which is frequently made in conventional regression models, is not required. In addition, the CART method allows for naturally incorporating interactions between predictors beyond what had previously been known, and these predictors can be easily interpreted by researchers.

These results highlight the ease with which CART models incorporate complex interactions. The interactions describe above would not have been detected by regression models, even with large sample sizes as the one in our study. Our CART was developed with 46 events and 5 predictors. When developing clinical prediction models with a binary outcome, a recommended sample size of 10 events per predictor is an extended rule [29–31]. Moreover, the internal validation of the CART provided by the bootstrap analysis showed that the results were very stable even with less than 4 % of events in the sample. In addition, CART models can handle missing values in a more natural way than more traditional techniques, in which subjects with missing values are eliminated from the analysis. In our decision tree, a clinician can extrapolate the likely mortality risk for a patient with missing information.

Our study has several strengths. The large sample and number of hospitals included reflect the general population of eCOPD patients evaluated in EDs in Spain. The decision tree uses measures generally gathered by physicians in the evaluation of eCOPD patients, and allows clinicians to easily establish prognosis without having to memorize the scores of different variables. It is much easier to use than complex and often cumbersome models. A similar CART model has been developed to evaluate the long-term (5 year) prognosis of patients with stable COPD. [5] Finally, our study points out an important issue as it is that mortality after 30 days after an ED visit is importantly conditioned by the comorbidities of the patient. This is important when planning the care and follow up of these patients.

Limitations of our study must also be noted. First, did not identify the causes of mortality. This could be important because cardiac events frequently occur after an eCOPD and could be related to previous cardiac disease rather than the eCOPD. The main limitation of decision tree models is that including higher-order interactions without considering the main effects could lead to spurious relations between predictors and overestimate the effect of some predictors. This is sometimes referred to as estimation bias. However, we believe that the use of combined split and bootstrap validation techniques provides internally and externally validated results that minimize spurious relations, as has been shown in previous studies [6]. Our tree is proposed to be used at the ED, or even before at the primary care level when seeing a eCOPD patient, as a decision making tool. Nevertheless, since our algorithm include either admitted to the hospital and discharge to home patients a bias should be taken into account since more fatalities were obviously found among patients admitted to the hospital. Therefore, caution must be taken if the algorithm is used exclusively with patients already admitted or discharged home. As an additional limitation of the study we must include that no inter or intra observer reliability studies were performed. Nevertheless, reviewers were trained by the principal investigators at each site and were provided with a manual for the collection of data.

## Conclusions

In summary, a CART model based on measures commonly collected in the ED evaluation of patients experiencing an eCOPD created a simple decision tree that identifies patients’ risk of short-term (30/60-day) mortality. Use of this decision could provide valuable information about the appropriate clinical care for these patients.

## Additional file

Additional file 1: eTable S1. Descriptive statistics stratified by sample, derivation vs. validation. **eTable S2.** Internal validation of the CART analysis by bootstrap resampling (*N* = 2000). **eFigure S1.** Results of internal validation of the CART analysis by bootstrap resampling (N=2000). (DOCX 36 kb)
