# Supplementary material for: A decision tree to assess short-term mortality after an emergency department visit for an exacerbation of COPD: a cohort study
Source: Respir Res. 2015 Dec 22;16:151. doi: 10.1186/s12931-015-0313-4 (PMC4699373; doi:10.1186/s12931-015-0313-4)

eTable 1. Descriptive statistics stratified by sample, derivation vs. validation.

|  |  | | |
| --- | --- | --- | --- |
|  | **Derivation**  **1252 (50.3%)** | **Validation**  **1235 (49.7%)** | **p-value** |
| Age* | 72.7 (9.8) | 72.9 (9.5) | 0.5532 |
| Sex (Male) | 1147 (91.6) | 1123 (90.9) | 0.5466 |
| Baseline FEV1% |  |  | 0.5799 |
| ≥50 | 42 (4.0) | 34 (3.3) |  |
| 30< FEV1%<50 | 301 (28.8) | 310 (30.2) |  |
| ≤30 | 701 (67.2) | 681 (66.4) |  |
| Charlson Comorbidity Index* | 2.24 (1.56) | 2.29 (1.60) | 0.4879 |
| >1 | 737 (58.9) | 747 (60.5) | 0.4103 |
| Diabetes mellitus | 257 (20.6) | 275 (22.5) | 0.2523 |
| Cardiopathy | 369 (29.5) | 355 (28.7) | 0.2936 |
| Previous LT-DOT or NIMV at home | 414 (33.1) | 427 (34.6) | 0.4268 |
| Number of admission in the previous year due to eCOPD* | 0.85 (1.38) | 0.82 (1.37) | 0.4891 |
| 0-1 | 1005 (80.3) | 1003 (81.2) | 0.4683 |
| 2 | 105 (8.4) | 110 (8.9) |  |
| ≥3 | 142 (11.3) | 122 (9.9) |  |
| Glasgow Coma Scale score <15 | 39 (3.1) | 31 (2.5) | 0.3618 |
| Heart rate upon ED arrival (≥120) | 112 (9.0) | 125 (10.1) | 0.5984 |
| Use of accessory inspiratory muscles upon ED arrival | 279 (22.3) | 231 (18.7) | 0.027 |
| Paradoxical breathing upon ED arrival | 61 (4.9) | 50 (4.1) | 0.3200 |
| pH upon ED arrival |  |  | 0.2694 |
| ≥7.35 | 1016 (87.8) | 975 (85.7) |  |
| 7.26-7.34 | 144 (9.9) | 136 (12.0) |  |
| <7.26 | 27 (2.3) | 27 (2.4) |  |
| PCO2 upon ED arrival |  |  | 0.9876 |
| ≤45 | 623 (57.1) | 609 (57.3) |  |
| 46-55 | 247 (22.6) | 237 (22.3) |  |
| 56-65 | 120 (11.0) | 121 (11.4) |  |
| >65 | 101 (9.3) | 96 (9.0) |  |
| MRC breathlessness scale |  |  | 0.1486 |
| 1-2 | 406 (32.4) | 382 (30.9) |  |
| 3 | 230 (18.4) | 271 (21.9) |  |
| 4 | 340 (27.2) | 332 (26.9) |  |
| 5 | 138 (11.0) | 138 (11.2) |  |
| Missing | 138 (11.0) | 112 (9.1) |  |
| Hospital admission | 782 (62.5) | 755 (61.1) | 0.4961 |
| Death within 30 days of ED evaluation | 46 (3.7) | 42 (3.4) | 0.7122 |
| Death within 60 days of ED evaluation | 79 (6.3) | 76 (6.2) | 0.8721 |
| *Represented as mean (std) |  |  |  |

eTable 2. Internal validation of the CART analysis by bootstrap resampling (*N* = 2000).

|  | CART | Bootstrap resampling | |  |
| --- | --- | --- | --- | --- |
| Node | Observed Mortality risk | Estimated median mortality risk | 95% coverage interval | Risk group |
| 1 | 0 | 0 | -- | Low |
| 2 | 0.0215 | 0.0222 | (0.0095 – 0.0581) | Low |
| 3 | 0.0105 | 0.0105 | (0.0033 – 0.0240) | Low |
| 4 | 0.0202 | 0.0211 | (0.0090 – 0.0526) | Low |
| 5 | 0.0370 | 0.0368 | (0.0084 – 0.0741) | Medium |
| 6 | 0.0816 | 0.0816 | (0.0213 – 0.1698) | High |
| 7 | 0.0435 | 0.0430 | (0.0106 – 0.0909) | Medium |
| 8 | 0.0818 | 0.0804 | (0.0323 – 0.1350) | High |
| 9 | 0.1846 | 0.1818 | (0.0882 – 0.2832) | Very high |
| 10 | 0.5556 | 0.5625 | (0.2222 – 0.8892) | Very high |

Estimated median mortality risk, 95% coverage intervals and stratification of risk are shown by node.

eFigure 1. Results of internal validation of the CART analysis by bootstrap resampling (N=2000).


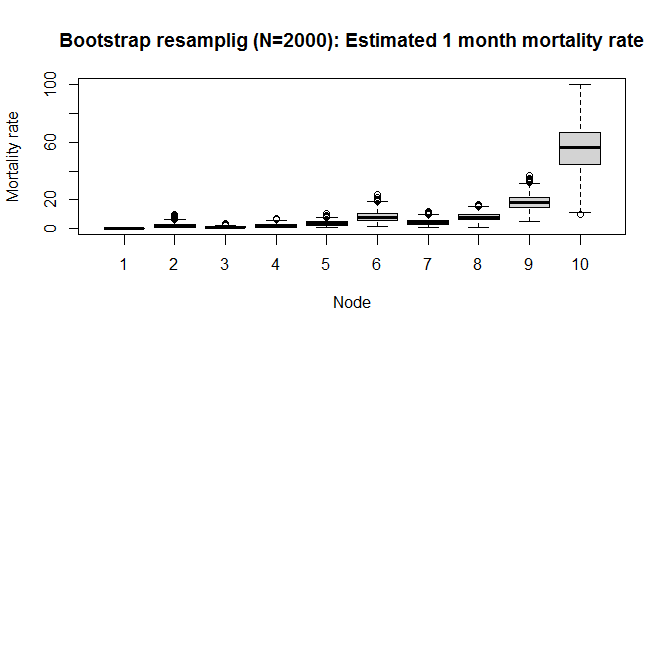

Supplement: Additional file 1: eTable S1. — Descriptive statistics stratified by sample, derivation vs. validation. eTable S2. Internal validation of the CART analysis by bootstrap resampling (N = 2000). eFigure S1. Results of internal validation of the CART analysis by bootstrap resampling (N=2000). (DOCX 36 kb) [file 12931_2015_313_MOESM1_ESM.docx]
